# Disparities in cancer epidemiology and care delivery among Brazilian indigenous populations

**DOI:** 10.1590/S1679-45082016AO3754

**Published:** 2016

**Authors:** Pedro Nazareth Aguiar, Gustavo Trautman Stock, Gilberto de Lima Lopes, Michelle Samora de Almeida, Hakaru Tadokoro, Bárbara de Souza Gutierres, Douglas Antônio Rodrigues

**Affiliations:** 1 Universidade Federal de São Paulo, São Paulo, SP, Brazil; 2 Centro Paulista de Oncologia, São Paulo, SP, Brazil; 3 Universidade Paulista, São Paulo, SP, Brazil

**Keywords:** Epidemiology, Public health, Ethnicity and health, Neoplasms, Health services accessibility, Brazil

## Abstract

**Objective:**

To assess aspects related to cancer in indigenous population.

**Methods:**

This is a retrospective study developed in a public university hospital. We included patients with 18 or more years of age, diagnosed with solid tumors, and followed between 2005 and 2015. Clinical features were assessed by descriptive statistics, and survival was evaluated by Kaplan-Meier curves and multivariate Cox regression.

**Results:**

Fifty patients were included. The cancer incidence was 15.73 per 100,000. The mean age at diagnosis was 54 years and most patients were female (58%). Cancer of the cervix (28%) and prostate (16%) were the most common. The mean time between the onset of symptoms and the diagnosis was 9 months and from diagnosis to the treatment was 3.4 months. Disease diagnosed at stage IV (17%) had worse overall survival (HR: 11.4; p<0.05). The 5-year survival rate ranged from 88% for prostate cancer to 0% for lung cancer. All 5-year survival rates were lower as compared to other populations.

**Conclusion:**

The most prevalent cancer sites were cervix and prostate. Disease stage and primary site were prognostic factors.

## INTRODUCTION

The former marked predominance of communicable diseases (CD) in developing countries has been rapidly replaced by emerging non-communicable diseases (NCD) in recent decades. Non-communicable diseases, once more frequent in high-income countries, are emerging as the leading cause of death globally.^(1,2)^

Cancer is the second most prevalent NCD, with 14 million new cases and 8.2 million deaths worldwide in 2012. These rates are expected to rise to an alarming 22 million new cases and 13 million deaths annually over the next two decades.^(3,4)^ Developed countries have the highest cancer rates; however, contrary to widely held beliefs, more than two-thirds of cancer related deaths occur in low- and middle-income countries, and are typically premature and preventable deaths.^(5)^ Behavioral risk factors and chronic infections account for at least 35% of cancer cases.^(5)^

According to the *Instituto Nacional de Câncer* (INCA) estimates, 420 thousand new cases of cancer will be diagnosed in 2016 (non-melanoma skin cancer excluded), with estimated incidence rates of 200 new cases per 100,000 individuals.^(6)^ Prostate, lung and colorectal cancer were the most prevalent types in Brazilian men, with 61.82, 17.49 and 16.84 new cases per 100,000 individuals, respectively (28.6%, 8.1% and 7.8% of all neoplasms reported in men). Breast, colorectal and cervical cancer were the most frequent types in Brazilian women, with 56.20, 17.10 and 15.85 new cases per 100,000 individuals, respectively (28.1%, 8.6% and 7.9% of all neoplasms reported in women).^(6)^

Recent international debates addressing indigenous health issues have brought the need to reduce disparities between indigenous and non-indigenous health to light.^(7)^ Despite high levels of intergroup heterogeneity, indigenous groups are among the most vulnerable segments of society worldwide, and are over-represented in groups living in extreme poverty and in disadvantaged and marginalized populations in their country of residence.^(8–10)^ Cancer prevalence data for indigenous populations are scarce; still estimates indicate rising incidences of the disease in indigenous communities.^(11)^ Evidence suggests lower cancer rates in indigenous people. However, significantly higher mortality rates as compared to the general population were reported.^(12,13)^ Adverse outcomes are thought to reflect advanced cancer stage upon diagnosis, lack of awareness of the disease, increased incidence of comorbidities and disparities in access to cancer care services.^(14)^

## OBJECTIVE

To investigate demographic aspects, disease characteristics and treatment outcomes and safety in a group of indigenous patients diagnosed as cancer.

## METHODS

### Patients

The sample in this study comprised indigenous patients seen at the Indigenous Health Department of the *Universidade Federal de São Paulo* (UNIFESP), Brazil, between January 2005 and May 2015. This project was developed in partnership with the Brazilian government. Patients with 18 or more years of age with histologically confirmed diagnosis of solid cancer were included in the study, regardless of treatment. Patients with benign tumors (five) or hematologic malignancies (three) were excluded. In Brazil, 34 Health Districts are in charge of providing Primary Care to indigenous populations according to geographical distribution. Further diagnostic and therapeutic demands are met by the UNIFESP Indigenous Health Department in nine of these districts. Therefore, all patients in this sample were referred from their respective Health District to UNIFESP Indigenous Health Service.

### Study design

Quantitative retrospective study based on data extracted from medical records, with the approval of UNIFESP Ethics Committee protocol number 1.462.985; CAAE: 40846314.4.0000.5505. Data were analyzed by two physicians with experience in cancer care and clinical research. Missing data in electronic medical records were obtained from the Medical Charts Storage (SAME - *Serviço de Arquivo Médico*) and Indigenous Health Support House (CASAI - *Casa de Apoio à Saúde do Índio*) where indigenous patients were housed over the course of treatment.

This study complied with resolution 466/12 of the Brazilian Ministry of Health. Overall survival of indigenous cancer patients was the primary endpoint; secondary endpoints were most common cancer types, and disparities in cancer epidemiology and care delivery between indigenous and non-indigenous citizens.

### Outcomes

Overall survival was defined as the time (months) between diagnosis and death, regardless of cause. Progression-free survival (PFS) was defined as the time (months) between implementation of treatment and the first radiologic evidence of disease progression or death, whichever occurred first. Time (months) from first symptoms to diagnosis and access to health care and from diagnosis to treatment was investigated. Response rate was based on investigators' assessment. Frequency of radiological screening was determined according to recommendations of the respective protocols (every 2 or 3 months, in general). Image analysis was based on Response Evaluation Criteria in Solid Tumors (RECIST) guidelines, version 1.1. Adverse events were graded according to Common Terminology Criteria for Adverse Events (CTCAE), version 4.

### Statistical analysis

Demographic characteristics were analyzed using descriptive statistics to determine frequencies, measures of central tendency and proportions.

Demographic data were categorized according to gender and compared using the χ^2^ (categorical variables) or the Student´s *t* test (numerical variables).

Despite potential selection biases in this study, the incidence rates of different types of cancer and overall cancer incidence rates in women and men were defined as the number of cases divided by age standardized at-risk population of the nine Health Districts covered by UNIFESP. Incidence rates were then compared with the estimated cancer incidence of the Indigenous People living in the United States for the period 2002 to 2006, and with the Brazilian and American overall population, in 2016.^(6,15,16)^

Overall survival and PFS were evaluated using the Kaplan-Meier method. Potential survival risk factors, such as lack of treatment, disease stage, cancer site and age were assessed using multivariate Cox regression. The level of significance was set at 5% (one-sided p value <0.05).

## RESULTS

### Demographic characteristics

Fifty adult patients diagnosed as cancer were seen at UNIFESP Indigenous Health Department between 2005 and 2015 (Table 1). Patient age ranged from 18 to 88 years (mean age, 54±16 years); 58% of patients were female; most patients were from the Southeast and Midwest regions of the country (48% and 38%, respectively). Data regarding smoking habits were available in 37 medical records; 18 out of 37 (47%) patients were smokers. Alcoholism was mentioned in 5 out of 30 (17%) medical records evaluated only. Thirty-four patients had some comorbidity, hypertension being the most prevalent (15 out of 50 patients; 30%); 8 (16%) patients were diabetic and 4 (8%) had a history of tuberculosis. Body mass index (BMI) data were available in 26 records; BMI ranged from 16.2 to 43kg/m^2^ (mean BMI, 25.7kg/m^2^).

**Table 1:** Baseline clinical and demographic characteristics in Indigenous patients

		Sex		p value
		Male (n=21)	Female (n=29)	
Age, years (n=50)				
	Mean	57.4	52.2	0.731
	Minimum	18	26	
	Maximum	77	88	
	SD	17.2	16.0	
Country region, % (n=50)				
	Southeast	47.6	48.3	0.530
	Midwest	38.0	37.9	
	North	14.3	6.9	
	Northeast	0.0	6.9	
Ethnicity, % (n=50)				
	Tupi-Guarani	14.3	17.2	0.558
	Pankararu	23.8	13.8	
	Kaiabi	9.6	10.3	
Smoking, % (n=50)				
	Yes	42.9	31.0	0.862
	No	38.1	37.9	
	NA	19.0	31.0	
Alcoholism, % (n=50)				
	Yes	23.8	0.0	0.008
	No	38.1	58.6	
	NA	38.1	41.4	
BMI, kg/m^2^ (n=26)				
	Mean	23.5	26.9	0.834
	Minimum	20.9	16.2	
	Maximum	28.0	43.0	
	SD	2.5	7.4	
Comorbidities, % (n=50)				
	Hypertension	38.1	24.1	0.675
	*Diabetes mellitus*	14.3	17.2	
	Pulmonary tuberculosis*	4.8	10.3	
Time from first symptoms to service arrival, months (n=36)				
	Mean	9.9	9.2	0.707
	Minimum	2.0	0.7	
	Maximum	42.3	32.1	
	SD	10.4	8.9	
Time from service arrival to diagnosis, months (n=29)				
	Mean	3.3	1.4	0.356
	Minimum	0.1	0.1	
	Maximum	10.7	8.4	
	SD	3.3	2.0	
Time from first symptoms to diagnosis, months (n=36)				
	Mean	9.7	8.6	0.533
	Minimum	2.5	0.5	
	Maximum	42.4	32.3	
	SD	10.4	7.4	
Time from diagnosis to first treatment, months (n=34)				
	Mean	4.9	2.5	0.003
	Minimum	0.0	0.0	
	Maximum	23.2	8.2	
	SD	6.5	2.3	

* Previously treated.

SD: standard deviation; NA: not assessed; BMI: body mass index.

### Cancer care delivery

Cancer care delivery data were available in 36 medical records. Time from first cancer symptom to access to health services ranged from 0.7 to 42.3 months (mean time: 9.4±9.6 months). Twenty-nine cases were diagnosed at UNIFESP; time to diagnosis ranged from 0.1 to 10.7 months in these cases (mean time: 2.3±2.9 months). Overall, time from first cancer symptom to diagnosis ranged from 0.5 to 42.4 months (mean time: 9±8.8 months). Fifteen patients (30%) did not receive treatment; of these, eight were not eligible for treatment and received only the best possible support care and seven refused treatment on cultural and religious grounds. Time from diagnosis to treatment ranged from zero to 23.2 months (mean time: 3.4±4.6 months).

### Epidemiological features

**Figure 1:**
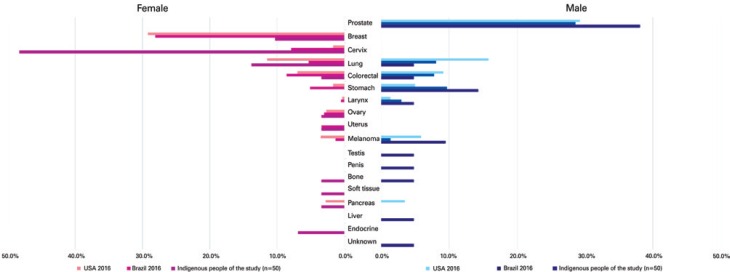
Most common cancer sites

The distribution of cancer types in the sample studied was as follows: cervical (14 cases; 28%), prostate (8;16%), lung (5;10%), non-colorectal gastrointestinal (5;10%), breast (3;6%) and colorectal (2;4%). Eight out of 48 (17%) patients were diagnosed with advanced stage (stage IV) disease. Cancer types and comparative data between the general Brazilian and United States populations are displayed on figure 1.

**Figure 2:**
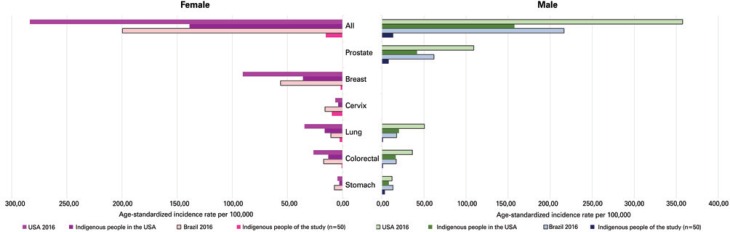
Cancer incidence rates

Overall cancer incidence rates in the sample studied was 15.73 cases per 100,000 individuals (15.19 and 13.28 in women and men, respectively). Cervical (9.71 per 100,000), prostate (7.78 per 100,000) and stomach cancer (male patients; 3.33 per 100,000) accounted for the highest incidence rates. Cancer incidence rates and comparative data between the general Brazilian population and indigenous and general United States populations are displayed on figure 2.

### Outcomes

Median follow-up time, PFS and overall survival were 32.2, 30.1 and 81.4 months, respectively.

**Figure 3:**
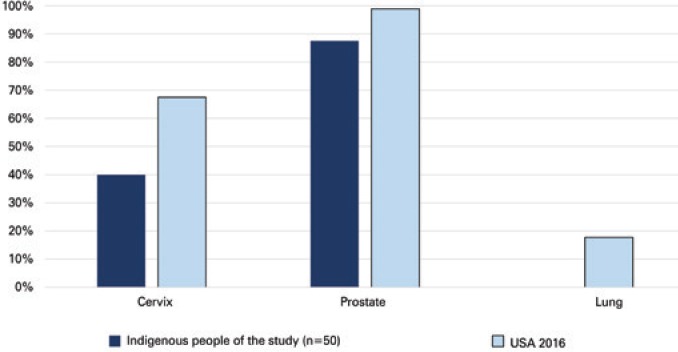
Five-year survival rates

Five-year survival rate differed according to primary cancer site, ranging from 88% to 0% (prostate and lung cancer respectively). However, overall cancer survival rates in this study were lower than 5-year survival rate estimates from United States populations (Figure 3).

Multivariate Cox regression analysis confirmed the primary site of cancer and disease stage upon diagnosis as negative prognostic factors for overall survival.

**Figure 4:**
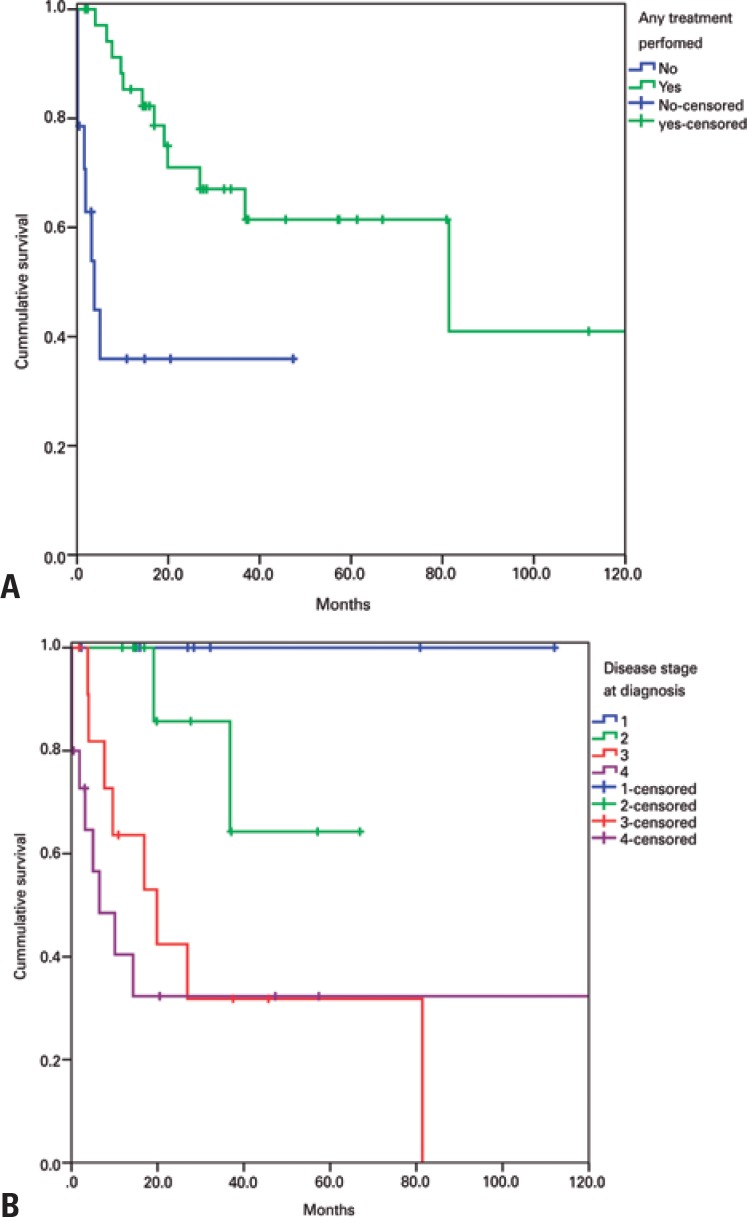
(A) Overall survival of treated and non-treated patients. (B) Overall survival according to disease stage at diagnosis

Median overall survival of non-treated patients (15 patients; 53% not eligible for treatment and 47% refusing treatment) was limited to 3.8 months. Lack of treatment was a non-significant predictor of poor survival (HR: 5.25; 95% confidence interval –95%CI: 0.81–34.10; p=0.08) (Figure 4A). Advanced stage at diagnosis was a significant prognostic factor (HR: 11.44; 95%CI: 1.39–94.23; p=0.024) (Figure 4B). Patients affected with lung cancer had the lowest median overall survival (6.5 *versus* 36.8 months and 81.4 months for cervical and prostate cancer respectively; HR: 12.11; 95%CI: 1.19–122.94; p=0.008). Sixteen patients received palliative chemotherapy or curative radiotherapy. Response to treatment data were available in 13 medical records and revealed 30.8% response rate. Platinum salts were the most commonly prescribed chemotherapeutic agents (8 patients; 50% of those receiving chemotherapy).

### Safety

Cytotoxic agents were generally well tolerated. The total number of cycles was 92 (1 to 16 cycles; mean 6.1). Most patients (75%) experienced adverse events, but less than half were graded 3 to 4. Neutropenia graded 1 to 2 or 3 to 4 was documented in 13.3% and 26.7% of patients, respectively. Grade 1 to 2 anemia was reported in 26.7% of patients. Dose reduction was not required in any of the cases studied; discontinuation of treatment due to adverse events was limited to two cases.

## DISCUSSION

Evidence obtained from several studies based on cancer registry data show that cancer is one of the leading causes of death in non-indigenous people, particularly in developed countries. Lower cancer incidence rates and poorer outcomes in indigenous compared to non-indigenous populations living in the same country were also reported.^(17)^

Communicable diseases continue to be a significant cause of morbidity and mortality in developing countries, particularly in low income communities. However lifestyle changes driven by economic development and globalization led to concurrent NCD emergence.^(18)^ This overlapping phenomenon (referred to as double disease burden) reflects the current epidemiologic transition scenario in many indigenous communities worldwide.^(19)^

Studies involving Amazon Indians from the Parkatejê community revealed that closer contact with civilization promoted behavioral and social changes among indigenous people; progressive transition from hunting habits and game based diets to sedentary lifestyle and high-fat diets led to increased overweight and obesity rates, and higher prostate cancer risks.^(20)^

Brazil has one of the smallest percentages of indigenous people within Latin America; still indigenous ethnicities vary widely.^(10)^ According to the national 2010 demographic census, indigenous communities comprised nearly 896,900 people (approximately 0.47% of the general population).^(21)^

The paucity of data on indigenous people overall health status is a significant limiting factor in assessment of indigenous health needs.^(15)^ Most reports to date refer to specific indigenous groups and respective health issues, and reveal significant health inequalities.^(10)^

According to national studies investigating mortality rates among indigenous people living in the state of Rio Grande do Sul (RS), lung, cervical and stomach cancer rank third, seventh and ninth as leading causes of death in this group.^(22)^ In contrast, studies involving the Suyá people living in the Xingu Indigenous Park revealed that cervical cancer ranked fourth as cause of death, from 1970 to 2004.^(23)^

Surprisingly, no cases of breast cancer were reported in Terena indigenous women.^(24)^ Prevalence of protective factors in remote communities, such as early age at first pregnancy, multiparity, and prolonged breastfeeding, may account for low breast cancer incidence rates compared to non-indigenous women.

Ethnicity-specific data provided by the Brazilian population-based cancer register of INCA are limited to Recife (PE), Manaus (AM), Curitiba (PR), Brasília (DF), Salvador (BA) and the state of Roraima (RR).^(25)^ Age-adjusted incidence rates per 100,000 individuals tend to be 10 to 50% lower in indigenous compared to non-indigenous people in all locations, with the exception of male and female patients living in Salvador (BA) and Recife (PE), respectively, regardless of cancer type*.* ^(25)^ Still, heterogeneous data rendered comparisons difficult.^(25)^ Prostate cancer was more common in men living in Salvador (BA), Brasília (DF) and Curitiba (PR), while stomach cancer prevailed in men living in Roraima (RR), Recife (PE) and Curitiba (PR).^(25)^ Cervical cancer ranked first in women living in Roraima (RR) and Manaus (AM), second in women living in Brasília and Curitiba and third in those living in Recife (PE) and Salvador (BA).^(25)^

Recent studies based on data from population-based cancer registries and including 24,815 cancer cases diagnosed in indigenous people living in Australia, New Zealand, Canada and the United States, between 2002 and 2006, revealed overall burden of cancer up to 50% lower in American, approximately 20% lower in Australian and less than 10% lower in Canadian indigenous people compared to their non-indigenous counterparts.^(15)^ In contrast, the overall cancer burden was 20 to 30% higher in indigenous people living in New Zealand and Alaska.^(15)^ Lung, prostate and colorectal cancer were the most common types of cancer in indigenous men; breast cancer was more common in indigenous women in most locations, followed by lung, colorectal and cervical cancer.^(15)^ The incidence of cervical cancer was higher in indigenous compared to non-indigenous women in several regions.^(15)^

Cancer type distribution in this study was similar to data given in the literature. However, cancer incidence rates differed widely. This may have been due to the potential selection bias introduced by the fact that only cancer patients referred from their respective local Health Districts for treatment at a university health reference center were included in the sample. Some patients may have moved to a different district for personal reasons, then received treatment at different health centers. Also, cancer screening may be underestimated in Indigenous Health Districts. Therefore, cancer incidence rates in this study may have been underestimated.

High incidences of cervical cancer among indigenous women may be attributed to precocious sexual activity, multiple sexual partners, high prevalence of sexually transmitted diseases, such as human papillomavirus (HPV) infection, and low coverage of Pap smear test and HPV vaccination.^(15,26)^ Specific risk factors for gastric cancer in indigenous people include tobacco use and/or exposure to indoor smoke, alcohol consumption and *Helicobacter pylori* infection.^(27)^ High rates of chronic viral hepatitis and alcohol abuse may account for the high incidence of hepatocellular carcinoma.^(27)^

Comprehensive indigenous people health studies from Australia, New Zealand and North America show that indigenous people with cancer tend to be diagnosed at a more advanced stage and have unfavorable outcomes.^(11,12,14,28)^

Cultural differences and health beliefs may affect the relation between indigenous patients and healthcare professionals, with negative impacts on indigenous patients' cancer care experience.^(29)^ Such mismatches may foster mistrust and prevent the implementation of appropriate treatment. The fact that indigenous patients may have a different perception of their disease must be taken into account for proper recognition of cultural obstacles and therapeutic preferences.^(29)^

Expansion of cost-effective strategies, such as cancer prevention programs and educational campaigns, may contribute to increased indigenous people awareness of cancer and respective modifiable risk factors via promotion of healthier eating habits, regular physical activity, abstention from tobacco smoking and alcohol abuse and avoidance of indoor pollution from biomass burn.^(27)^ Intense surveillance of environmental contaminants such as pesticides, heavy metals, and industrial waste in the vicinity of indigenous lands is also recommended to prevent further exposure to carcinogens.^(27)^

The vaccination of indigenous people against HPV and hepatitis B virus may translate into decreased incidences of cervical and hepatocellular cancer in the middle term.^(27)^ The impact of *Helicobacter pylori* eradication on gastric cancer risks in indigenous populations remains open to debate, particularly in the light of questionable feasibility in remote communities; still, treatment of high-risk groups may be warranted.^(30)^

Screening tests, such as Pap smears, are vital for early diagnosis and treatment of precancerous and cancerous lesions and have major positive impacts on cancer incidence and mortality.^(27)^ However, potential benefits depend on the extent of coverage, quality of cytology collection and analysis, patient compliance and access to treatment and follow-up. A large proportion of the indigenous population lives in rural and remote areas; therefore, well-orchestrated referral to secondary and tertiary cancer care centers is a major factor in provision of specialized treatment to patients affected with advanced stage disease.^(27)^

Optimization of local primary healthcare services and incorporation of skilled, culturally competent health care providers sensitive to indigenous people beliefs and needs may help to overcome cultural gaps.

Indigenous populations have been relentlessly fighting for equity in healthcare. Disparities in cancer outcomes between indigenous and non-indigenous people are rooted in several profound, complex historic factors. There is still much to be done in order to develop appropriate cancer prevention strategies for indigenous people and to improve their access to cancer diagnosis and treatment.

## CONCLUSION

Cervical and prostate cancers were the most prevalent types in the sample studied. Primary site of cancer and stage of the disease at diagnosis were the most significant prognostic factors. Further studies are warranted to confirm these data and promote the development of strategies aimed at improving cancer care among indigenous people.
